# Regulation of Synaptic Plasticity and Adaptive Convergence Under Rhythmic Stimulation of an In Vitro Hippocampal Neuronal Network of Cultured Cells

**DOI:** 10.3390/bios16010065

**Published:** 2026-01-19

**Authors:** Shutong Sun, Longhui Jiang, Yaoyao Liu, Li Shang, Chengji Lu, Shangchen Li, Kui Zhang, Mixia Wang, Xinxia Cai, Jinping Luo

**Affiliations:** 1 State Key Laboratory of Transducer Technology, Aerospace Information Research Institute, Chinese Academy of Sciences, Beijing 100190, China; sunshutong23@mails.ucas.ac.cn (S.S.); jianglonghui22@mails.ucas.ac.cn (L.J.); liuyaoyao20@mails.ucas.ac.cn (Y.L.); shangli23@mails.ucas.ac.cn (L.S.); luchengji19@mails.ucas.ac.cn (C.L.); lishangchen24@mails.ucas.ac.cn (S.L.); zhangkui191@mails.ucas.ac.cn (K.Z.); wangmixia@mail.ie.ac.cn (M.W.); 2 School of Electronic, Electrical and Communication Engineering, University of Chinese Academy of Sciences, Beijing 100049, China

**Keywords:** in vitro microelectrode arrays, synaptic plasticity, rhythmic modulation, adaptive regulation

## Abstract

Synaptic plasticity constitutes a fundamental mechanism of neural systems. Rhythmic activities (e.g., θ and γ oscillations) play a critical role in modulating network plasticity efficiency in biological neural systems. However, the rules governing plasticity and adaptive regulation of in vitro cultured networks under structured electrical stimulation remain insufficiently characterized. To quantitatively investigate these regulatory effects within a highly controlled and low-interference environment, we utilized primary mice hippocampal neurons cultured on multielectrode arrays (MEAs) and executed two dedicated sets of experiments. (1) Spatiotemporal electrical stimulation paradigms, combined with connectivity analysis, revealed pronounced regulation effects of network plasticity. (2) Physiologically inspired rhythmic stimulation (θ: 7.8 Hz, γ: 40 Hz) with varying pulse repetitions was then applied. Although both rhythms induced distinct frequency-dependent plasticity modulation, the disparity between their modulatory effects progressively diminished with increasing stimulation pulse numbers, suggesting an intrinsic adaptive regulatory mechanism. Collectively, our findings characterize the effects of plasticity regulation and reveal the mechanisms underlying adaptive convergence in in vitro neuronal systems. These results advance the understanding of network plasticity, providing a technical foundation for functional shaping and modulation of in vitro neural networks while supporting future explorations into learning-oriented modulation.

## 1. Introduction

Synaptic plasticity is a fundamental mechanism of neural systems. By modulating the strength of synaptic connections between neurons, it enables dynamic information regulation at the network level [1]. In neuronal culture systems, the collective manifestation of synaptic plasticity is referred to as network plasticity, which forms the foundation for learning and memory functions in biological neuronal networks (BNNs) [2].

**Figure 1:**
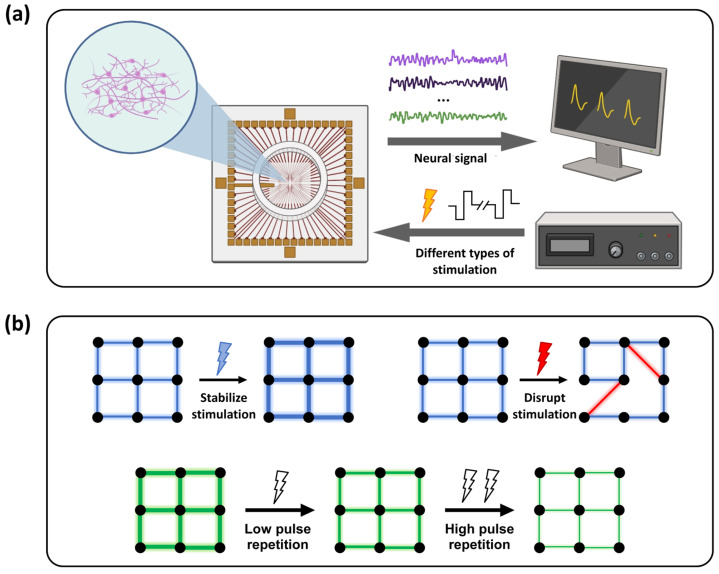
Schematic diagram of the in vitro neuronal networks platform and electrical stimulation protocol. (**a**) Electrophysiological detection and electrical stimulation platform based on an in vitro microelectrode array (MEA). Hippocampal neurons were cultured in the medium within the circular area of the MEA surface. (**b**) Experimental scheme of electrical stimulation. The first row illustrates the regulatory effects of high- and low-frequency electrical stimulation on network functional connectivity. The blue lightning represents the stabilization effect stimulation paradigm, while the red lightning represents the disruption effect stimulation paradigm; The second row depicts the modulation of neuronal firing patterns under rhythmic stimulation of different pulse repetitions levels. The number of lightning indicates the level of pulse repetitions.

In vitro neural network models offer unique advantages for investigating synaptic plasticity and the fundamental regulatory mechanisms under highly controlled conditions. These systems allow for precise control of external inputs and eliminate individual variability and non-task-related neural interference [3]. Based on multi-electrode array (MEA) technology, in vitro neural networks preserve fundamental neuronal and synaptic properties and enable noninvasive, high temporal- and spatial-resolution detection of electrophysiological activities [4]. Additionally, MEA platforms support programmable multi-site electrical stimulation, providing an ideal environment for investigating the formation, organization, and modulation of neuronal populations [5], as well as stimulus-induced network dynamics (Figure 1a) [6,7]. Consequently, these systems facilitate the study of fundamental synaptic mechanisms, such as plasticity, within controlled neural cell cultures.

The spike timing dependent plasticity (STDP) framework provides a fundamental model for understanding synaptic regulation: when presynaptic spikes precede postsynaptic spikes, synaptic strength is potentiated; the reverse sequence leads to synaptic depression [8]. Moreover, high-frequency electrical stimulation can induce long-term potentiation (LTP) [9], whereas low-frequency stimulation tends to elicit long-term depression (LTD) [10]. Changes in synaptic strength are reflected in neuronal firing patterns at the population level. Specifically, strengthened effective synaptic coupling induces enhanced synchrony within particular firing patterns, thereby elevating functional connectivity metrics.

Learning behavior is tightly coupled to synaptic plasticity. By modulating synaptic efficacy, STDP significantly enhances the learning efficiency of BNNs [11]. Moreover, during learning processes, neuronal firing activity often evolves toward structured and task-specific synchronous patterns [12]. Previous studies have utilized frequency-dependent plasticity to apply distinct electrical stimulation patterns in in vitro neuronal cultures, successfully achieving task-oriented modulation of network activity that exhibits learning- and memory-like behaviors [13,14,15,16]. However, due to variations in BNN characteristics, learning objectives, and encoding-decoding strategies, it is challenging to establish a clear mapping between stimulation methods and changes in synaptic structure within the network [2]. Therefore, it is necessary to further investigate the regulatory principles of network plasticity in highly controlled in vitro neural models with minimal internal interference.

Additionally, there remains a notable lack of systematic studies that explore the dynamic evolution of network plasticity within in vitro BNNs under physiologically inspired rhythmic modulation [17]. Consequently, inspired by the critical roles of θ and γ oscillations in neural processes [18,19], rhythmic stimulation at representative frequencies can be applied in MEA-based in vitro neural cultures. This approach may enable a systematic investigation of the regulatory principles underlying network plasticity of in vitro neural cultures.

Building upon this foundation, this study developed an in vitro MEA platform on which cell cultures derived from the mice hippocampus were cultivated. Primary hippocampal neurons were chosen specifically due to their well-documented high plasticity potential [20,21], serving as a canonical and reliable in vitro model for investigating the fundamental rules of synaptic plasticity and collective synaptic interactions. Using the signal acquisition and stimulation capabilities of multi-electrode arrays, we systematically investigated the effects of different spatiotemporal stimulation paradigms on network connectivity, confirming that in vitro hippocampal BNNs exhibit plasticity modulation characteristics, providing a mechanistic foundation for exploring adaptive regulatory mechanisms. Furthermore, we applied physiologically inspired rhythmic electrical stimulation at 7.8 Hz (the fundamental Schumann Resonance frequency, which is closely linked to nervous system functions [22,23], and falls within the hippocampal θ band) and 40 Hz (a prototypical γ frequency). By systematically modulating the repetitions of stimulation pulse, we quantitatively investigated the dynamic regulation of network firing patterns under varying degrees of repeated stimulation (Figure 1b). The experimental results demonstrated that, as the number of rhythmic stimulation pulses increased, the originally distinct frequency-dependent regulatory differences within the network significantly diminished. This finding reveals the frequency-specific characteristic of adaptive convergence within the neuronal network and its dynamic evolution as a function of pulse repetitions.

In the MEA-based in vitro hippocampal neural network model, this study systematically characterized the regulatory principles governing functional network connectivity, and demonstrating the capacity of plasticity within the network. Furthermore, we quantitatively characterized the frequency-specific adaptive regulatory properties of neuronal networks under rhythm-inspired stimulation with different pulse repetition levels. Overall, our work provides fundamental insights into network plasticity regulation and the regulation effects of adaptive convergence. Moreover, these results provide experimental support and a methodological framework for the functional shaping and modulation of in vitro neural networks, potentially supporting future implementations of learning-oriented modulations.

## 2. Materials and Methods

### 2.1. Reagents and Apparatus

Phosphate-buffered saline (PBS, 0.1 M, pH 7.4) and sodium glutamate (≥99%) were procured from Shanghai Chemical Reagent Company (Shanghai, China). HBSS, DNase, papain, DMEM, and Neurobasal Plus Medium were supplied by Sigma Aldrich (St. Louis, MO, USA), while cytarabine, and Polyl-D-Lysine was obtained from Thermo Fisher (Waltham, MA, USA). The MEA interfaces were modified using an electrochemical workstation (Gamry Reference 600, Gamry Instruments, Warminster, PA, USA). Electrophysiological recordings were conducted with a 128-channel acquisition system from Blackrock Microsystems (Salt Lake City, UT, USA). Additional instruments used throughout the experiments included a scanning electron microscope (Hitachi, Ltd. Tokyo, Japan), preamplifier (Blackrock Microsystems), a CO_2_ incubator (Thermo Fisher), a dual-channel stimulator (Multichannel. Reutlingen, Germany), and an oscilloscope (TPS2024, Tektronix, Beaverton, OR, USA).

### 2.2. Fabrication of In Vitro Microelectrode Arrays and Neuron Cultivation

**Figure 2:**
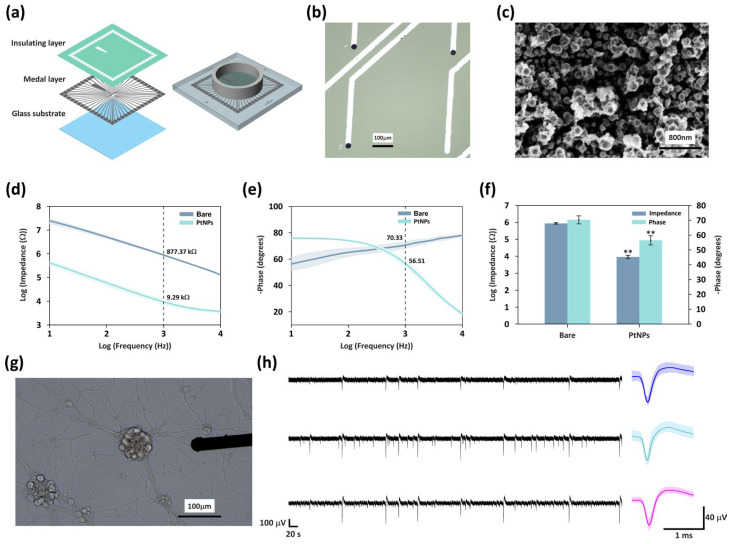
Fabrication and Modification of MEA. (**a**) Illustration of the structure of the MEA. (**b**) Optical microscopy image of bare site and PtNPs modified sites (scale bar: 100 µm). (**c**) SEM image of PtNPs deposited on electrode sites (scale bar: 800 nm). (**d**) Impedance characteristic of PtNPs-modified sites and bare sites across various frequencies. (**e**) Phase characteristic of PtNPs-modified sites and bare sites across various frequencies. (**f**) Mean impedance and phase at 1 kHz for unmodified and PtNP-coated sites. ** indicates *p* < 0.01 (Mann–Whitney U test, *n* = 5 per group), where n denotes the number of independence electrodes. For both parameters, *p* = 0.008, *U* = 0, but demonstrated a large effect size (*r* = 0.83). (**g**) Growth state of mice hippocampal neurons on the MEA surface (scale bar: 100 μm). (**h**) Raw data of one-minute spontaneous electrophysiological signals from three representative active recording sites (scale bars: 100 µV, 20 s). The corresponding averaged action potential waveforms (mean ± SD), extracted from the high-frequency components, are presented on the right (scale bars: 40 µV, 1 ms).

To facilitate the investigation of regulatory principles of network plasticity and adaptive mechanisms within in vitro hippocampal cell cultures under electrical stimulation, an electrophysiological MEA platform was established. We designed and fabricated the custom MEA using microelectromechanical systems (MEMS) surface micromachining technology. As shown in Figure 2a, the MEA architecture comprises a glass substrate for mechanical support and structural stability, a conductive metallic layer for signal routing, and an insulation top layer. Within this framework, 60 circular electrode sites (30 μm diameter) are uniformly arranged in a square grid at the chip center with a 600 μm pitch, following previous MEA designs [24,25]. Central electrode sites are connected to peripheral bonding pads via micro-wires, enabling interfacing with external acquisition systems. Detailed fabrication steps and parameters are provided in Figure S1.

To enhance the electrical performance and biocompatibility of the electrodes, the MEA sites were modified with platinum nanoparticles (PtNPs) via Chronoamperometry using a two-electrode system. The electrodeposition was conducted at a step potential of −0.85 V for 20 s. Owing to their excellent physicochemical stability, large specific surface area, and superior electrical conductivity, PtNPs can significantly improve the electrodes’ performance in both electrophysiological signal detection and stimulation delivery [26]. Consequently, the PtNPs-modified MEA exhibits excellent functionality and reliability in neuronal signal acquisition and electrical stimulation applications. The detailed PtNP modification procedure is provided in Text S1.

In this study, hippocampal tissue was selected for in vitro neuronal culture. All animal procedures were approved by the Institutional Animal Care and Use Committee of the Aerospace Information Research Institute, Chinese Academy of Sciences, and conducted in strict accordance with the guidelines of the Beijing Association for Laboratory Animal Care (AIRCAS-006-1, 14 September 2022). All surgical procedures were performed following established ethical standards to minimize animal suffering. In this study, pregnant mice at embryonic day 15.5 were euthanized, and the uterus was removed for subsequent hippocampal tissue isolation and culture. Neuronal cultures were prepared following previously established protocols [27,28]. The seeding density was 2000 cells/mm^2^. The detailed procedures for tissue isolation and culture are provided in Text S2.

### 2.3. Modulation of Network Connectivity Under Distinct Electrical Stimulation Paradigms

**Figure 3:**
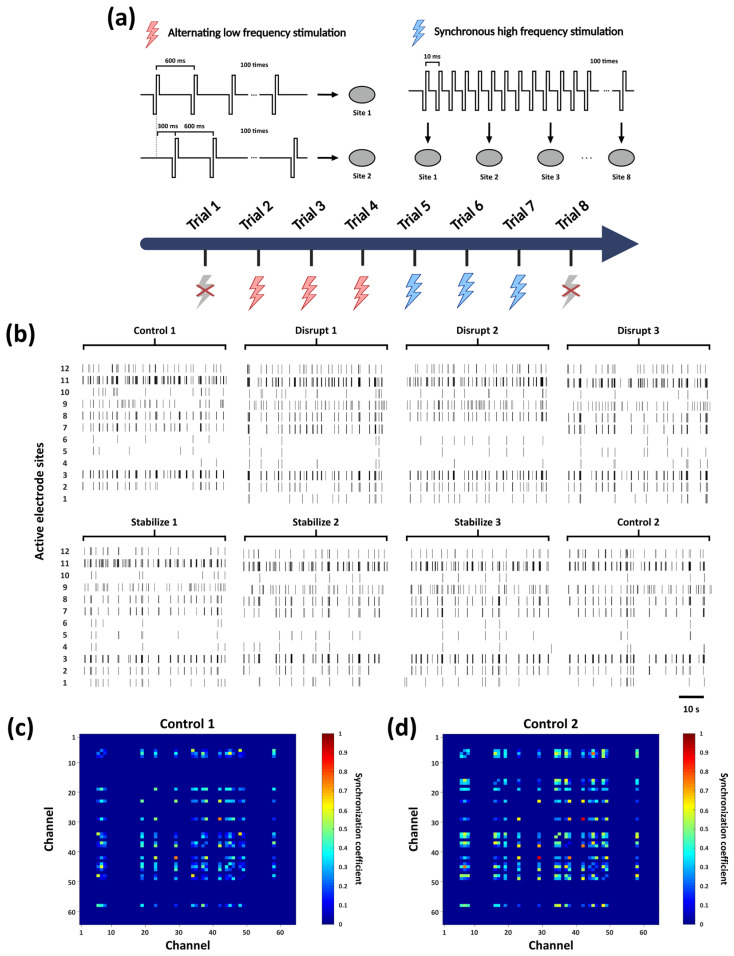
Analysis of network firing activity under a series of electrical stimulations. (**a**) Schematic of the stimulation experiment: gray lightning denotes the absence of electrical stimulation, red lightning represents low-frequency “Disrupt” stimulation applied alternately between two electrodes at 300 ms intervals, while blue lightning represents, high-frequency 100 Hz “Stabilize” stimulation applied synchronously to eight electrodes on the MEA. Each stimulation trial consisted of 100 pulses, with three consecutive “Disrupt” trials followed by three “Stabilize” trials. (**b**) The spike firing patterns recorded in the first minute of each trial are shown, with only the most active sites displayed in the figure. (**c**) Synchrony heatmap of firing nodes of “Control 1” trial which conducted before the electrical stimulation trials. (**d**) Synchrony heatmap of firing nodes of “Control 2” trial which conducted after the electrical stimulation trials.

To investigate whether the in vitro hippocampal neuronal networks in this study exhibit frequency-dependent plasticity at the network level, two representative stimulation paradigms were developed (Figure 3a): “Disrupt” and “Stabilize”.

The “Disrupt” paradigm aimed to weaken pre-existing network connectivity. Its design was motivated by prior evidence demonstrating that low-frequency stimulation induces long-term depression (LTD), thereby leading to an attenuate of synaptic connectivity. Prior studies have employed low-frequency stimulation protocols, such as 1 Hz and 5 Hz, to reshape neuronal network connectivity [13,14]. Furthermore, paired stimulation delivered via fixed electrode pairs has been shown to effectively drive plasticity changes in shared pathways [29,30]. Accordingly, our “Disrupt” protocol employed asynchronous pulses at 300 ms intervals (~3.3 Hz) between two specific electrodes to achieve the disruption of functional connectivity.

The “Stabilize” paradigm was designed to enhance network stability. High firing rates and regular synchronous activity are established catalysts for inducing long-term potentiation (LTP) [31,32,33]. Previous studies have demonstrated that predictable high-frequency stimulation, such as 100 Hz, can strengthen synaptic connectivity within neuronal networks [14]. Accordingly, our “Stabilize” protocol employed 100 Hz high-frequency synchronous stimulation across eight evenly distributed electrodes on MEA to enhance network stability.

Both paradigms utilized negative-phase-precedence biphasic pulses (amplitude: ±300 mV, width: 200 μs) with 100 pulses per trial. This configuration ensures charge neutrality through biphasic compensation, thereby preventing electrode polarization and electrochemical damage [34]. Based on the impedance of the PtNPs-coated electrodes (~9 kΩ, Figure 2f) and the site diameter (30 μm), the estimated charge per phase is 6.67 nC, yielding a geometric charge density of 0.94 mC/cm^2^ [35]. This value is well within the safe operating limits for nanostructured platinum electrodes (e.g., a reported maximum of 3.48 mC/cm^2^ for 17 kΩ PtNPs-coated MEA electrodes) [36]. Furthermore, according to the Shannon criterion, the calculated k value is approximately 0.7. This value remains significantly below to the biological damage threshold of 1.5 [37], confirming the electrochemical and biological safety of the protocol.

Following each trial, electrophysiological signals were recorded continuously for 3 min for subsequent analysis, with the first and the last trials serving as blank control without stimulation. To eliminate potential short-term dependence or transient elastic changes induced by specific stimulation patterns [38], the experiment was conducted with three consecutive “Disrupt”–“Stabilize” trial cycles. This design enabled a systematic evaluation of how different stimulation paradigms modulate network connectivity. The stimulation paradigm is provided in Table S1.

To more clearly elucidate the impact of electrical stimulation on network firing dynamics, we calculated the Pearson correlation coefficients between spike signals recorded from all electrode pairs, thereby quantifying neuronal synchrony (details provided in Text S3). Furthermore, to analyze network connectivity, we employed the Louvain community detection algorithm, which exhibits both high efficiency and accuracy in identifying modular structures within large-scale networks [39]. Based on the graph theory approach, this algorithm enables systematic analysis of neuronal clusters on the MEA, classifying and aggregating firing nodes into distinct subnetworks [40]. Specifically, the algorithm was applied to the synchrony coefficient matrix to segment the network into functional subnetworks, enabling quantitative comparisons of connectivity changes across different trials.

Subsequently, the subnetwork affiliations of individual node derived from the Louvain algorithm were integrated into a unified sequence to characterize the functional connectivity of the entire network. The Levenshtein edit distance was then used to quantify structural similarity between trials. As a classical sequence alignment algorithm, the Levenshtein distance has significant theoretical and practical value in biological sequence alignment and database similarity analysis [41]. Detailed implementations are provided in Text S4.

### 2.4. Modulation of Neuronal Firing Patterns by Rhythmic Stimulation with Varying Pulse Repetitions

To investigate the adaptive regulatory characteristics of in vitro neural cultures under rhythmic electrical stimulation, this study was inspired by the critical roles of θ and γ oscillations in learning and memory, selecting 40 Hz and 7.8 Hz as representative stimulation parameters. In the experimental design, a blank control group was first established, followed by three stimulation groups. Each stimulation group consisted of two trials, during which rhythmic electrical stimulation sequences at 40 Hz or 7.8 Hz were synchronously delivered to the entire network. A recovery interval of 10–20 min was provided following each stimulation trial. The pulse repetitions level of each stimulation group was adjusted based on the number of pulse repetitions in each trial: 100 pulses for Group 1, 1000 for Group 2, and 10,000 for Group 3 (the pulse characteristics are consistent with the method described in Section 2.3). The stimulation paradigm is provided in Table S2. After each trial, electrophysiological activity was continuously recorded for 3 min for subsequent analysis. To quantify the overall network state, we defined a network synchronization activity index as the product of the total number of functional connections and their average synchronization coefficient.

### 2.5. Electrophysiological Analysis

In this study, the sampling frequency for data acquisition was set to 30 kHz to accurately capture the neuronal firing events. The recorded raw data was subsequently analyzed using Offline Sorter (v3) and Neuroexplorer (v4). The Valley-Seeking algorithm was employed to extract the spike signals recorded at each site. The spike signals from each site are considered a sequence of spikes from the neurons. Subsequent analyses of all data were conducted using MATLAB 2018b (MathWorks) and Python 3.9 (Python Software Foundation). Key Python packages employed in this study included numpy, scipy, community, networkx, Levenshtein and matplotlib. Statistical analysis utilized both the Wilcoxon signed-rank test (for paired comparisons) and the Mann–Whitney U test (for independent comparisons). Corresponding effect sizes (r) were calculated and reported to quantify the magnitude of the observed differences.

## 3. Results

### 3.1. Enhanced Electrical Performance of MEA via Modification

Following the MEMS-based fabrication process (see Section 2.2), we successfully developed a high-density MEA platform for neural interfacing. As shown in Figure 2a, the device integrates 60 microelectrodes within a PDMS-defined culture chamber, providing a stable microenvironment and reliable electrical coupling for the long-term maintenance and recording of hippocampal networks in vitro.

In addition, platinum nanoparticles (PtNPs) were applied to modify the electrode sites of the MEA to enhance their electrical performance and biocompatibility. Compared with unmodified electrodes, the PtNPs-modified electrodes exhibited a darker surface appearance (Figure 2b). Scanning electron microscopy (SEM) revealed that PtNPs were distributed on the electrode surface, forming a hierarchical granular morphology (Figure 2c) that markedly increased the effective surface area, thereby providing a more extensive interface for neuronal adhesion.

The electrochemical characteristics of the modified electrodes were assessed using electrochemical impedance spectroscopy (EIS) in phosphate-buffered saline (PBS), with measurements performed across frequencies ranging from 10 Hz to 10 kHz (Figure 2d–f). The results showed that, compared to the bare electrodes, PtNPs modification significantly reduced the impedance at 1 kHz (the typical frequency reflecting neuronal firing characteristics) from 877.4 ± 89.8 kΩ to 9.3 ± 2.3 kΩ, representing a 94-fold decrease. Meanwhile, the phase delay decreased from 70.3 ± 2.7° to 56.5 ± 3.2°. The substantial reduction in impedance enhanced efficiency of charge transfer between the electrode and the biological tissue, and effectively improved the signal-to-noise ratio (SNR) of electrophysiological recordings. Meanwhile, the decreases in phase delay increases both the response speed and the accuracy of electrical signal transmission, collectively led to a substantial improvement in the overall electrical performance of the electrodes.

After culturing mice hippocampal neurons on the PtNPs-modified MEA for 21 days, the optical microscopy image revealed well-formed synaptic connections among neurons, indicating the initial establishment of a network structure (Figure 2g). We recorded the spontaneous activity of the network. The left panel of Figure 2h presents the raw electrophysiological signals obtained from three representative channels, demonstrating a high SNR (5.3172 ± 2.4816). We subsequently extracted the high-frequency components from each channel (band-pass filtered between 250 Hz and 5 kHz), and the corresponding average spike waveforms are shown on the right. further confirming the excellent capability of this MEA platform for high-quality multichannel neural signal detection.

### 3.2. Modulation of Network Firing Synchrony Following Electrical Stimulation Trials

Using the electrical stimulation protocol described in Section 2.3, we applied two stimulation patterns (“Disrupt” and “Stabilize”) to the in vitro biological neural networks, with a total of eight trials performed, as shown in Figure 3a. The raster plots in Figure 3b illustrate the spike activity recorded from a subset of active electrodes within one minute following each stimulation trial. In the initial “Control 1” trial (prior to stimulation), synchronous firing events were already observed at several active sites, suggesting that neurons near these electrodes may serve as key nodes for information transmission within the network. Across multiple stimulation trials, the firing patterns exhibited discernible variations. Moreover, after the full sequence of stimulations, the firing synchrony observed in “Control 2” was further enhanced compared with “Control 1”.

Subsequently, we performed the firing synchrony analysis. As shown in Figure 3c, the synchrony heatmap of “Control 1” reveals the presence of initial functional connections among subsets of neurons prior to stimulation. In contrast, “Control 2” (Figure 3d) exhibited a marked increase in the number of synchronous firing nodes, indicating a substantial enhancement in overall network synchrony (synchrony analyses for all trials are provided in Figure S2). These findings indicate that the applied series of stimulation paradigms effectively enhanced the coordinated activity of the in vitro neural network and promoted the formation of functional connectivity patterns. Building on this observation, we then conducted a systematic investigation into the specific regulatory effects of the different stimulation paradigms on network functional connectivity.

### 3.3. Bidirectional Modulation of Network Functional Connectivity by Distinct Stimulation Paradigms

Based on the preceding firing synchrony analysis, the Louvain algorithm was applied to further characterize the modulation of network functional connectivity under distinct stimulation paradigms (Figure 3a).

**Figure 4:**
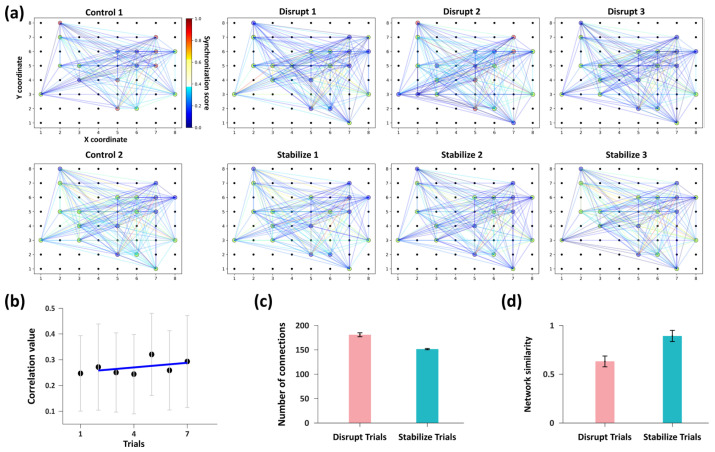
Analysis of network functional connectivity. (**a**) Eight subgraphs correspond to the network communication patterns observed in each trial shown in Figure 3a. In each graph, nodes of different colors represent distinct subnetworks (note that the node colors are used solely to distinguish subnetworks and do not indicate functional attributes; thus, node colors of identical subnetworks may vary across experimental groups). The connecting edges are weighted by the synchrony coefficients between nodes, representing the functional connections. (**b**) Variation trend of the average synchrony coefficient of active nodes across trials. (**c**) Comparison of the total number of functional edges among active nodes between two stimulation patterns. (**d**) Comparison of the average network similarity between the two stimulation patterns. Statistical analysis for both (**c**,**d**) are performed using the Wilcoxon signed-rank test (*n* = 3 per group, where n denotes the number of measurements pairs). For both parameters, *p* = 0.25, but demonstrated a large effect size (*r* = 0.93).

The visualization results revealed distinct differences in subnetwork organization and nodes connectivity were observed across trials as shown in Figure 4a. This suggests that the two stimulation paradigms exert clear modulatory effects on network connectivity. We therefore conducted further quantitative analyses to elucidate the specific regulatory influences of each stimulation mode on network connectivity.

We quantified the mean synchrony coefficient and the total number of functional connections among all active nodes in each trial. The “Control 1” trial exhibited the lowest network synchrony. Synchrony remained low during “Disrupt” trials, whereas “Stabilize” trials induced a slight increase, as indicated by a mild positive linear trend (Figure 4b). The number of active nodes remained stable across trials (Figure 4a); however, after “Disrupt” trials, the total number of functional connections between nodes (180.67 ± 4.03) was significantly higher than that observed after “Stabilize” trials (151.33 ± 1.25) (Figure 4c). These divergent responses indicates that the “Disrupt” paradigm destabilizes the functional architecture by inducing a large number of low-correlation links, reflecting a transition toward a disorganized and non-specific network stat. In contrast, the “Stabilize” paradigm refines the network, shifting it toward a more consolidated state characterized by higher firing synchrony and a streamlined set of stronger connections.

To further evaluate the specific regulatory effects of these two stimulation patterns on network connectivity, we quantified network similarity by applying the Levenshtein distance method to network connectivity sequences derived from subnetwork affiliations. We computed the network similarity between each trial and its preceding trial. Network similarity consistently remained low following each “Disrupt” stimulation, whereas it increased markedly after each “Stabilize” stimulation. Specifically, the mean similarity for “Disrupt” trials was 0.63 ± 0.06, in contrast to the substantially higher similarity of 0.89 ± 0.06 observed in “Stabilize” trials (Figure 4d).

These results demonstrate the capacity for network-level plasticity within our in vitro model. Consistent with the established LTD and LTP frameworks on synaptic regulation, the low-frequency asynchronous paired stimulation (“Disrupt” paradigm) effectively attenuates functional connectivity; conversely, the high-frequency synchronous stimulation (“Stabilize” paradigm) significantly enhances connectivity weights. Collectively, this bidirectional modulation confirms that the in vitro neural model exhibits intrinsic plasticity characteristics, providing an effective strategy and analytical framework for regulating functional connectivity in in vitro neuronal networks.

### 3.4. Modulation Effects of ISI Distribution and Firing Pattern Under Rhythmic Stimulation with Varying Pulse Repetitions

After confirming the robust plasticity characteristics of the in vitro hippocampal biological BNN, we further applied rhythmic stimulation with varying numbers of stimulation pulses to investigate the dynamics regulation processes of the network. Prior to each stimulation trial, the restoration of the fundamental firing state of the network was confirmed by monitoring the mean firing rate (MFR) during the recovery periods (Figure S3).

**Figure 5:**
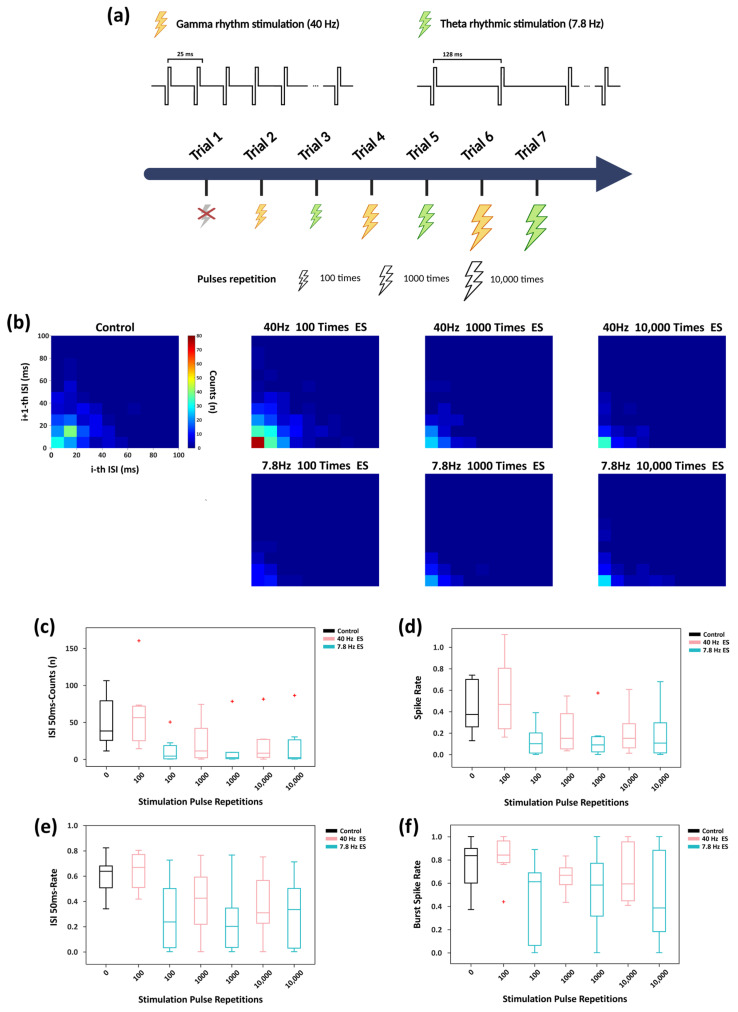
Characteristics of network firing dynamics under rhythmic stimulation of different pulse repetitions. (**a**) Schematic of the stimulation experiment: gray lightning denotes the absence of electrical stimulation, yellow lightning indicates γ-band (40 Hz) stimulation, while green lightning indicates θ-band (7.8 Hz) stimulation; both types of stimulation were applied synchronously to all electrode sites of the in vitro network. The two rhythmic stimulation trials were alternated, with stimulation repetition progressively increased by adjusting the pulses repetition per trial from 100 times to 1000 and then to 10,000. (**b**) Joint ISI distributions under various stimulation frequencies and pulse repetitions of rhythmic stimulation. (**c**) Boxplots showing the count of neural spikes occurring within a 50 ms window based on the joint ISI distribution. (**d**) Boxplot of firing rate in each trial. (**e**) Boxplot of proportion of firing events within 50 ms relative to the total events in each trial. (**f**) Boxplot showing the proportion of spikes that occur within bursts relative to all detected spikes (burst spike rate). In all boxplots, the whiskers extend to 1.5×IQR.

Following the protocol illustrated in Figure 5a and described in Section 2.4, the entire in vitro network was subjected to two representative oscillatory stimulation frequency, 40 Hz and 7.8 Hz, inspired by θ and γ rhythms, respectively. Joint interspike interval (ISI) distribution analyses (Figure 5b) revealed that at moderate stimulation repetition (100 pulses), 40 Hz stimulation markedly increased the concentration of short-interval firing events. In contrast, under the same stimulation repetition, 7.8 Hz stimulation substantially reduced short-interval firing events to levels lower than the control group. This initial frequency-dependent divergence indicates that higher-frequency stimulation initially induces a more pronounced tendency for short-interval firing events. However, this frequency-dependent difference is diminished under prolonged stimulation (≥1000-pulse repetitions), as short-interval firing events converge to slower, more similar levels between the two frequencies. Text S5 provides the calculation details for joint ISI distribution.

Further analysis (Figure 5c) showed that at 100-pulse repetitions, 40 Hz stimulation elicited substantially more high-density firing events within the 50 ms compared with the control condition. In contrast, 7.8 Hz stimulation suppressed these high-density occurrences, leading to a sparser and more desynchronized firing pattern relative to the control. As stimulation repetition increased, although 40 Hz stimulation still produced slightly more short-interval spikes than 7.8 Hz stimulation, overall short-interval activity was markedly reduced relative to the control, with the gap between the two frequencies gradually diminished. The firing-rate dynamics displayed a similar trend (Figure 5d).

To determine whether the reduction in short-interval events was solely attributable to a decrease in firing rate, we calculated the proportion of spikes occurring within 50 ms intervals in the joint ISI distribution (Figure 5e). At 100-pulse repetitions, 40 Hz stimulation significantly increased this proportion, whereas 7.8 Hz stimulation reduced it. With increased stimulation repetition, the difference between the two groups progressively decreased, and both stabilized at relatively low levels.

As shown in Figure 5f, burst spike rate analysis (calculation details provided in Text S5) further revealed that at 100-pulse repetitions, 40 Hz stimulation increased the burst spike rate relative to the control, whereas 7.8 Hz stimulation reduced it. At higher pulse repetitions, the burst spike rate under 40 Hz stimulation remained higher than that induced by 7.8 Hz stimulation at the same repetition level, but the difference between the two progressively diminished.

Taken together, these findings demonstrate that under relatively low pulse repetitions conditions (100-pulse repetitions), 40 Hz γ-rhythm stimulation more effectively enhances the overall firing rate and short-interval firing activity of the neuronal network compared with 7.8 Hz θ-rhythm stimulation, reflecting a characteristic frequency-dependent modulation of firing patterns. However, during prolonged stimulation (1000 and 10,000 pulse-repetition), the modulatory differences between the two frequencies diminished, with both groups exhibiting similar levels of network suppression. The regulatory effect of 7.8 Hz stimulation remained relatively stable across increased repetitions, whereas the 40 Hz-induced modulation exhibited a non-linear saturation.

### 3.5. Adaptive Convergence of Network Synchrony Activity Under Prolonged Rhythmic Stimulation

**Figure 6:**
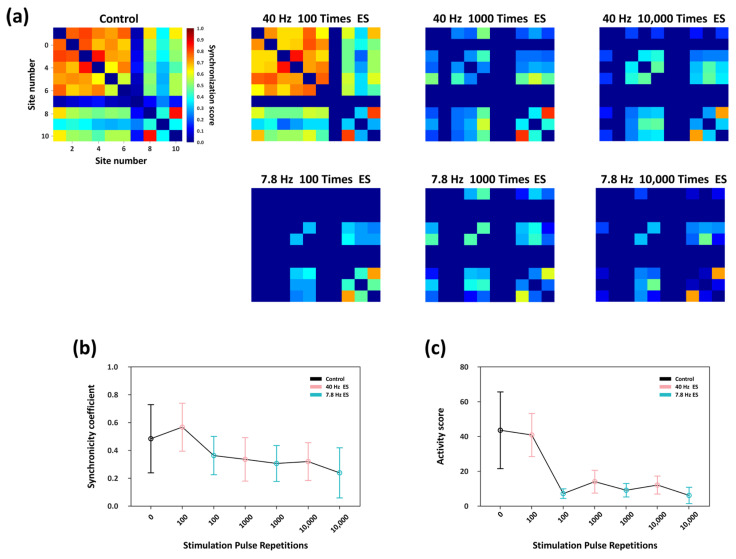
Network synchronization characteristics under rhythmic electrical stimulation of different pulse repetitions. (**a**) Heatmaps illustrating the firing synchrony among active nodes across experimental trials. (**b**) Variations in the average firing synchrony between active nodes under different stimulation conditions. (**c**) Changes in the network synchronization activity in each trial, defined as the product of the number of effective connecting edges and their mean connection weight.

Firing synchrony serves as a vital functional indicator for characterizing the network-level manifestations of synaptic plasticity. Accordingly, using the same correlation-based analytical framework described in Section 3.2, we quantitatively assessed the level of spike-train synchrony within the network under rhythmic electrical stimulation with different pulse repetitions levels. As shown in the heatmaps in Figure 6a, the neuronal cultures developed in vitro for 21 days had already formed relatively stable synaptic connectivity patterns; therefore, under spontaneous activity in the absence of external stimulation (Control trial), active nodes exhibited a baseline level of synchronous firing. Following 40 Hz stimulation with 100-pulse repetitions, synchrony among several key nodes was slightly enhanced relative to the control group, and the overall synchronization level remained comparable to that of the Control trial. In contrast, 7.8 Hz stimulation at the same repetition level markedly reduced network synchrony, with a visible decrease in the number of nodes exhibiting synchronous firing. When the stimulation pulse repetitions increased to 1000 pulse, 40 Hz stimulation continued to induce higher synchrony than 7.8 Hz, although the synchrony level was lower than that under the 100-pulse repetitions condition. Further increasing the repetition to 10,000 resulted in uniformly low synchrony for both stimulation frequencies, with no significant difference between them.

Figure 6b summarizes the changes in the average synchronization coefficient across effective nodes. Under 100 repetitions, 40 Hz stimulation significantly enhanced inter-node synchrony relative to the control condition, whereas 7.8 Hz stimulation reduced it. As stimulation pulse repetitions increased, the frequency-dependent difference in synchrony between the two stimulation conditions progressively diminished, and the overall synchronization level continued to decline. These results indicate that rhythmic stimulation with a high number of pulse repetitions not only suppresses global network synchrony but also substantially attenuates the intrinsic frequency-dependent regulatory differences observed between 40 Hz and 7.8 Hz stimulation.

Furthermore, we conducted the network synchronization activity analysis. As shown in Figure 6c, at a repetition of 100 pulses, 40 Hz stimulation produced no significant effect on this metric relative to the control trial, whereas 7.8 Hz stimulation produced a marked reduction. As the number of stimulation pulse repetitions increased, the network synchronization activity induced by 40 Hz stimulation showed a pronounced decrease, whereas this measure remained relatively unchanged under 7.8 Hz stimulation. Consequently, the difference in the modulation effect on network synchronization between the two frequencies became non-significant.

Taken together, these findings demonstrate that rhythmic stimulation at 40 Hz and 7.8 Hz exerts distinct frequency-dependent regulation effects on network synchrony. However, with increasing stimulation pulse repetitions, the regulatory effect of 40 Hz stimulation was significantly attenuated, while that of 7.8 Hz stimulation remained largely unchanged. Consequently, the initial frequency-dependent divergence narrowed, leading to a convergence of the regulatory outcomes between the two frequencies, and the overall level of network synchrony remains persistently low.

## 4. Discussion

In this study, a 60-channel microelectrode array was fabricated based on MEMS technology, and platinum nanoparticles were electrodeposited onto the electrode surface using a two-electrode configuration (Figure 2a–c). This surface modification markedly reduced electrode impedance and phase delay, thereby improving the electrical performance of the MEA (Figure 2d–g).

Primary hippocampal neurons derived from mice were cultured on the MEA platform for 21 days, allowing them to develop into networks randomly interconnected via synapses. Initially, the regulatory effects of various spatiotemporal electrical stimulation paradigms on the in vitro neural networks were evaluated. The low-frequency asynchronous pattern significantly decreased network structural similarity before and after stimulation, indicating disruption of pre-existing functional connectivity. In contrast, the high-frequency synchronous pattern maintained higher structural similarity, suggesting a stabilization of functional connectivity (Figure 4). These findings demonstrate that the in vitro neuronal network exhibits distinct plasticity characteristics, where different spatiotemporal configurations exert bidirectional regulatory effects on network connectivity. This establishes a foundation for subsequent experiments. Furthermore, it provides both an effective method for plasticity regulation based on functional connectivity and an analytical framework for characterizing network dynamics.

Furthermore, we applied physiologically inspired electrical stimulation at different representative rhythmic frequencies to investigate the dynamic regulation of network plasticity under varying levels of stimulation pulse repetitions (Figure 5 and Figure 6). Stimulation experiments were conducted using stimulation pulse repetitions of 100, 1000, and 10,000. In each experimental condition, synchronous electrical stimulation at 40 Hz and 7.8 Hz was applied. Under 100 pulses repetition level, 40 Hz stimulation markedly enhanced burst activity and firing synchrony within the network, whereas 7.8 Hz stimulation produced the opposite effect, demonstrating a clear characteristic of frequency-dependent plasticity regulation. In contrast, under higher pulse repetitions level (≥1000 repetitions), the frequency-dependent modulation was progressively attenuated, with the differences in regulation effects between the two frequencies no longer significant.

These phenomena can be partially explained by a habituation mechanism. Under prolonged stimulation, neural networks may develop cumulative effects, including dysregulation of neurotransmitter mobilization and utilization, ion channel blockade, and enhanced afterhyperpolarization. These changes lead to reduced neuronal excitability and weakened synaptic function [42,43]. Moreover, this adaptive suppressive effect exhibits frequency dependence, with higher stimulation frequencies inducing more pronounced inhibitory effects [44].

In this study, systematic analyses of key metrics—including inter-spike interval (ISI), firing rate, burst rate, and network synchrony—were conducted to evaluate stimulation-induced regulatory effects. The results showed that the responses to 7.8 Hz stimulation remained relatively stable, whereas those induced by 40 Hz stimulation weakened with increasing numbers of stimulation pulse repetitions. Correspondingly, the initially significant differences in regulatory effects between the two stimulation frequencies gradually diminished as pulse repetitions increased, transitioning from significant to marginal differences. These results further indicate that stimulation frequency and pulse repetitions jointly constitute critical factors governing the regulatory effects of adaptive convergence in in vitro neuronal networks.

Previous studies have reported that in the brain, memory processes often display a “γ-nested-in-θ” rhythmic pattern [45,46,47]. In this framework, moderate inhibition and rebound recovery prevent overexcitation and support the formation of stable memory ensembles [48]. This operational mode may be conceptually related to the frequency-specific adaptive regulation observed in our in vitro neural networks, suggesting that the brain can dynamically adjust the frequency and duration of neural activity to coordinate such adaptive regulatory effects, thereby maintaining a stable functional state. Furthermore, these findings provide inspiration for the long-term modulation of in vitro neuronal networks. The adoption of multi-frequency asynchronous or nested stimulation paradigms, analogous to the brain’s intrinsic adaptive balancing mechanisms, may enable more efficient, and stable network regulation.

The present findings indicate that at high pulse repetitions (1000 and 10,000), the regulatory efficacy of 40 Hz observed at 100 pulses was significantly attenuated, whereas the effects of 7.8 Hz remained largely unchanged. This suggests that the pulse repetitions threshold required to achieve inhibitory balance may vary across frequencies. Moreover, at pulse repetitions of 1000 and 10,000, the remaining marginal differences between the two conditions remained nearly unchanged. This observation suggests that the adaptive suppressive regulation may reach a saturation limit. Therefore, systematic exploration across a broader range of frequency–pulse repetitions combinations will be essential to uncover the principles and boundary conditions of frequency- and pulse repetitions-dependent adaptive regulatory mechanisms. Regarding the statistical analysis, we acknowledge that certain metrics did not achieve formal significance; however, the observed large effect sizes (0.80–0.90) indicate a substantial biological trend. This lack of significance is primarily attributable to the low statistical power inherent in small sample sizes. As an exploratory study, we recognize these statistical constraints. Future research will aim to expand the sample size to further validate and consolidate these preliminary findings.

Furthermore, we acknowledge that the quantitative assessment of the network recovery process was relatively insufficient in the current study, and the influence of cumulative stimulation load has not been explicitly excluded; although our rest periods (Figure S3) ensured the restoration of a baseline firing rate, the observed regulatory effects may still be affected by residual plasticity from the network’s history. To address these limitations, future work should incorporate a comprehensive counter-balanced design and combine this with a multi-culture independent experimental setup.

Additionally, it will be necessary to integrate electrochemical detection techniques with electrophysiological recordings to investigate neurotransmitter dynamics. This approach will not only reveal network architectures at the synaptic level with greater clarity, but also enable a more detailed investigation of the dynamic recovery process of neural networks under different stimulation paradigms. Together, these analyses will provide a more comprehensive and in-depth complement of plasticity modulation and adaptive convergence in neural networks.

## 5. Conclusions

This study first characterizes the response of in vitro cultured hippocampal biological neuronal networks on a multichannel microelectrode array under diverse spatiotemporal stimulation paradigms. Our results reveal distinct plasticity regulation patterns: while high-frequency synchronous stimulation effectively stabilized and strengthened the functional connectivity of network, low-frequency asynchronous stimulation exerts a disruptive effect. These findings confirm that the in vitro neuronal model possesses intrinsic plasticity regulatory characteristics, providing a mechanistic basis for investigating adaptive network regulatory effects. Moreover, they offer effective approaches for plasticity regulation and analysis of biological neuronal networks based on functional connectivity.

Building on these results, experiments utilizing rhythmic stimulation inspired by intrinsic brain rhythms were performed. The analyses showed that frequency-dependent modulation of network firing patterns progressively diminished as the number of stimulation pulse repetitions increased across different rhythms, ultimately leading to a convergence of regulatory effects. Our study quantitatively characterized the response of the in vitro neural networks to varying pulse repetitions, revealing the dynamic features of frequency-specific adaptive regulatory effects. These results provide crucial theoretical inspiration and experimental groundwork for the functional modulation of in vitro biological neural networks.

This study not only elucidates the fundamental network plasticity characteristics and their regulatory effects, but also uncovers the mechanism adaptive regulatory. These findings provide experimental evidence for understanding the regulatory principles that support learning processes in in vitro neuronal systems. Moreover, they establish a theoretical foundation for developing cross-frequency and cross-repetition plasticity modulation strategies.

## Data Availability

The raw data supporting the conclusion of this article will be made available by the authors, without undue reservation.

## Supplementary Materials

The following supporting information can be downloaded at: https://www.mdpi.com/article/10.3390/bios16010065/s1, Figure S1. Fabrication process of in vitro multi-channel microelectrode arrays. Figure S2. Heatmaps of spike synchrony within the network under distinct stimulation paradigms across trials. Figure S3. Mean firing rate of the network throughout the full experimental sequence. Table S1. Experimental paradigm for network connectivity modulation under distinct spatiotemporal stimulation protocols. Table S2. Experimental paradigm for modulation of network firing patterns by rhythmic electrical stimulation. Text S1. Details of Platinum Nanoparticles (PtNPs) modification. Text S2. Details of mice hippocampal tissue isolation and neuronal culture. Text S3. Algorithm for neuronal firing synchrony coefficient. Text S4. Network similarity analysis based on Levenshtein distance method. Text S5. Algorithm for joint ISI distribution. Text S6. Algorithm for burst statistics.
