# Supplementary material for: Regulation of Synaptic Plasticity and Adaptive Convergence Under Rhythmic Stimulation of an In Vitro Hippocampal Neuronal Network of Cultured Cells
Source: Biosensors (Basel). 2026 Jan 19;16(1):65. doi: 10.3390/bios16010065 (PMC12838577; doi:10.3390/bios16010065)
Supplement: Supplementary file 1 [file biosensors-16-00065-s001.zip › biosensors-4036721-supplementary.pdf]

Supplementary Materials

Regulation of Synaptic Plasticity and Adaptive Convergence under Rhythmic Stimulation of an In vitro Hippocampal Neuronal Network of Cultured Cells

Shutong Sun [1,2] Longhui Jiang [1,2] Yaoyao Liu [1,2] Li Shang[1,2] Chengji Lu [1,2] Shangchen Li [1,2] Kui Zhang [1,2] Mixia Wang [1,2] Xinxia Cai [1,2*] Jinping Luo [1,2*]

[1] State Key Laboratory of Transducer Technology, Aerospace Information Research Institute, Chinese Academy of Sciences, Beijing 100190, China
[2] School of Electronic, Electrical and Communication Engineering, University of Chinese Academy of Sciences, Beijing, 100049, China.
* Correspondence: Author: Jinping Luo, jpluo@mail.ie.ac.cn; Xinxia Cai, xxcai@mail.ie.ac.cn.

Supplementary Figure S1. Fabrication process of in vitro multi-channel microelectrode arrays.

Supplementary Figure S2 Heatmaps of spike synchrony within the network under distinct stimulation paradigms across trials.

Supplementary Figure S3 Mean firing rate of the network throughout the full experimental sequence.

Supplementary Table S1 Experimental paradigm for network connectivity modulation under distinct spatiotemporal stimulation protocols.

Supplementary Table S2 Experimental paradigm for modulation of network firing patterns by rhythmic electrical stimulation.

Supplementary Text S1 Details of Platinum Nanoparticles (PtNPs) modification

Supplementary Text S2 Details of mice hippocampal tissue isolation and neuronal culture

Supplementary Text S3 Algorithm for neuronal firing synchrony coefficient.

Supplementary Text S4 Network similarity analysis based on Levenshtein distance method

Supplementary Text S5 Algorithm for joint ISI distribution.

Supplementary Text S6 Algorithm for burst statistics.

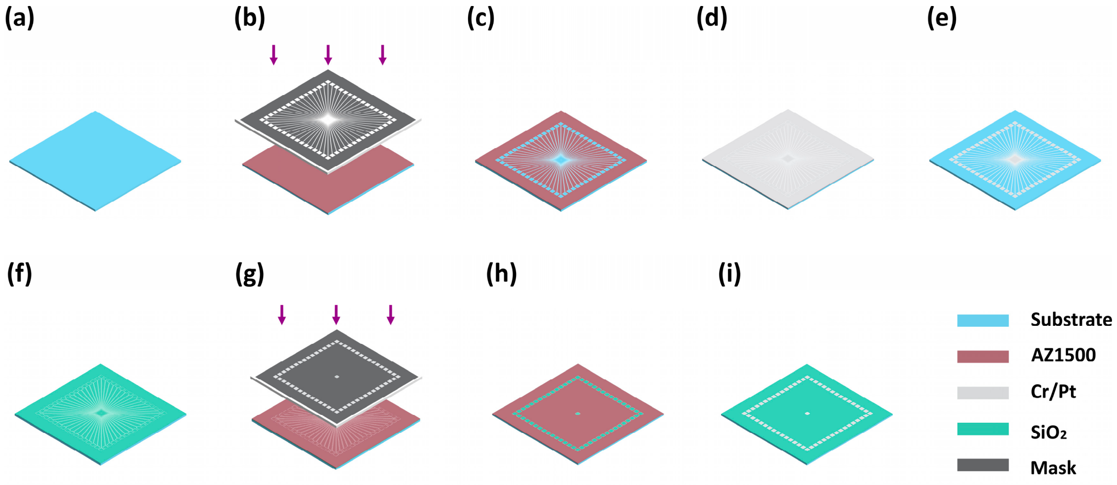

Figure S1. Fabrication process of in vitro multi-channel microelectrode arrays. (**a**) Glass substrate. (**b–e**) Photolithography was performed to transfer the metal layer pattern onto the photoresist AZ1500. (**c**) Development was performed to remove the exposed areas of the photoresist. (**d**) Metal layer deposition was carried out using sputtering. (**e**) Lift-off was performed to remove the metal on the residual photoresist. (**f**) The SiO_2 insulating layer was deposited using the PECVD method. (**g,h**) Photolithography and development process were performed to expose the regions of sites, reference electrode and pads. (**i**) The insulating layer on the sites, reference electrode and pads were removed using C_4H_8 reactive ion etching, followed by cleaning off the residual photoresist.

In this study, we utilizing MEMS technology to fabricate a 60-channel MEA to serve as an in vitro culture platform for neuronal cells. The detailed design of the MEA was based on previous work[1]. The complete process steps and details are described below. First, AZ1500 positive photoresist was uniformly spun on the surface of glass substrate cleaned with concentrated sulfuric acid. UV photolithography is then performed using the first photomask to transfer the pattern of conductive medal layer onto the photoresist, followed by development (Figure S1 a-c). Next, the medal layer (Cr: 30nm/Pt: 250nm) was sputtered and lifted off to create patterns for microelectrode sites, wires and pads on the substrate (Figure S1 d, e). Subsequently, an insulating layer (800nm SiO_2) was deposited via plasma-enhanced chemical vapor (PECVD) deposition (Figure S1 f). A second photolithography process, using the second photomask, was followed by reactive ion etching with C_4H_8 to expose the microelectrode sites, reference electrodes, and pads while maintaining other areas insulated (Figure S1 g-i).

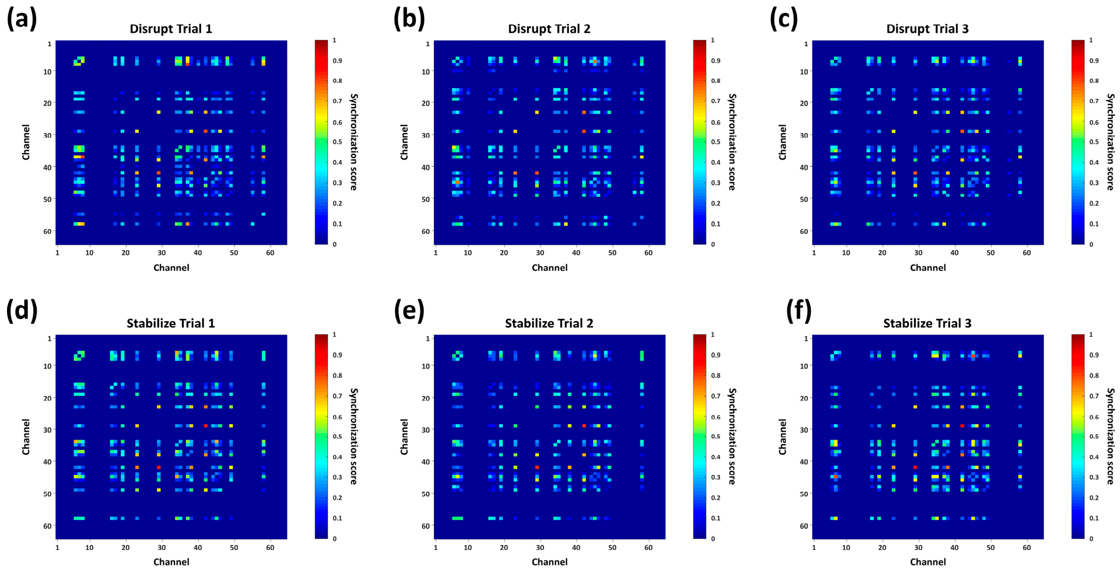

Figure S2. Heatmaps of spike synchrony within the network under distinct stimulation paradigms across trials.

Figure S2 a–f illustrates the network firing synchrony heatmaps obtained during electrical stimulation trials at different frequencies, as detailed in Table S1.

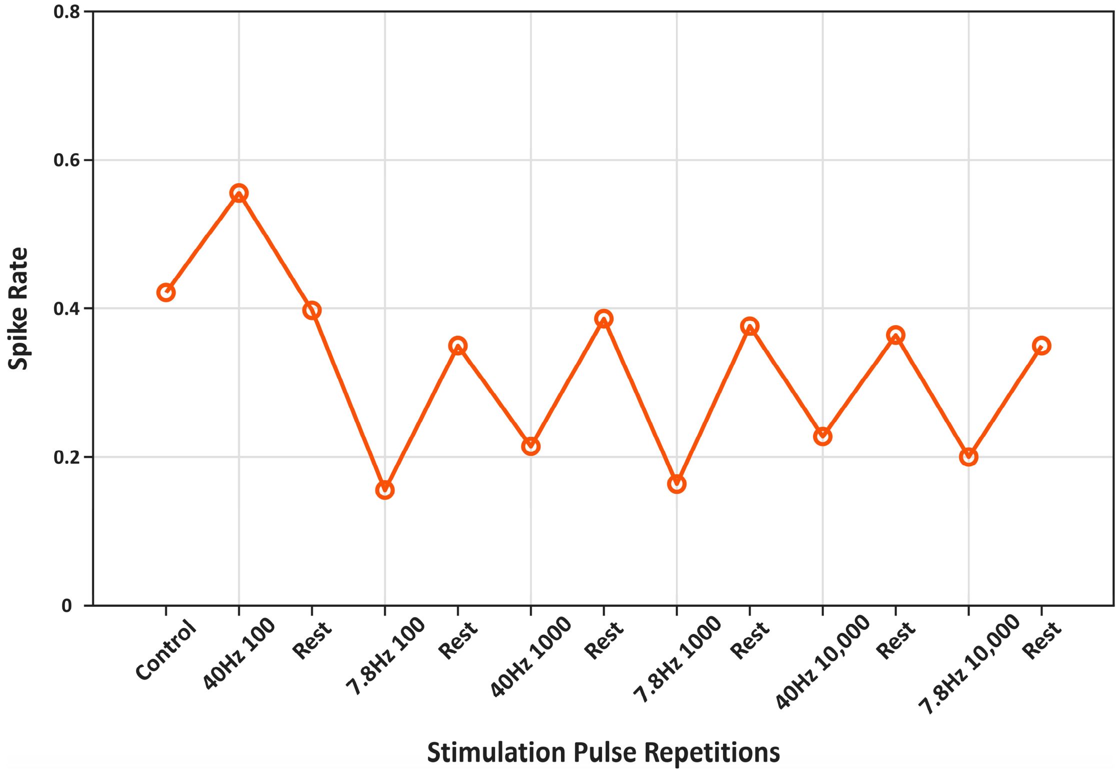

Figure S3. Mean firing rate of the network throughout the full experimental sequence.

Between sequential stimulation trials, an inter-trial interval of 10–20 min was implemented. Subsequent stimulations were performed after verifying that the firing state of active electrodes in the network had successfully recovered to control levels.

To visually demonstrate the evolution of network excitability throughout the entire experimental procedure, a time-course plot was employed to represent the mean firing rate (MFR) of active electrodes across each stimulation trial and its subsequent recovery phase. The results indicate that in the low-repetition group (100 pulses), the MFR exhibited transient fluctuations following 40 Hz and 7.8 Hz stimulations but showed a clear trend of returning to the initial control baseline. In contrast, in the high-repetition groups (1000 / 10,000 pulses), the firing rate significantly decreased after 40 Hz stimulation, converging toward the levels observed at 7.8 Hz. Notably, across all stimulation paradigms, the network firing activity consistently restored to a stable level comparable to the control group following the designated rest periods. These results confirm that the spontaneous firing activity returned to near-control levels after rest, suggesting a recovery of the basal firing level.

Table S1. Experimental paradigm for network connectivity modulation under distinct spatiotemporal stimulation protocols.

Stimulation trials	Intensity (Repetition count)	Recording duration
Control	/	3 min
Disrupt	100	3 min
Disrupt	100	3 min

Disrupt	100	3 min
Stabilize	100	3 min
Stabilize	100	3 min
Stabilize	100	3 min
Control	100	3 min

In the table, "Stimulation trials" indicate the stimulation paradigm applied in each trial, where "Control" denotes the absence of electrical stimulation, "Disrupt" refers to low-frequency alternating electrical stimulation, and "Stabilize" corresponds to high-frequency synchronous stimulation. "Intensity" represents the number of repetitions of voltage stimulation pulses within a given trial, while "Recording duration" specifies the period over which the network's electrophysiological activity is recorded following the stimulation.

Table S2. Experimental paradigm for modulation of network firing patterns by rhythmic electrical stimulation.

**Stimulation trials**	**Intensity (Repetition count)**	**Recording duration**
Control	/	3 min
40 Hz	100	3 min
7.8 Hz	100	3 min
40 Hz	1000	3 min
7.8 Hz	1000	3 min
40 Hz	10,000	3 min
7.8 Hz	10,000	3 min

In the table, *Stimulation trials* denote the stimulation paradigms applied in each trial, where *Control* indicates the absence of electrical stimulation, and *40 Hz/7.8 Hz* specify the frequencies of the electrical stimulation delivered to the network. The definitions of *Intensity* and *Recording duration* are consistent with those provided in Table S1.

Text S1 Details of Platinum Nanoparticles (PtNPs) modification

The electroplating solution for PtNPs was prepared by mixing chloroplatinic acid solution at a concentration of 48 mM with lead acetate solution at a concentration of 4.2 mM in a 1:1 ratio. PtNPs were then electroplated onto the surface of gold electrode sites using two-electrode system, with the electrode sites serving as the working electrode and Pt as the counter electrode immersed in the solution. Chronoamperometry was conducted on electrochemical workstation for the PtNPs deposition. The step potential was -0.85, sampling interval was 1s and duration was 20s.

Text S2 Details of mice hippocampal tissue isolation and neuronal culture

The neuronal cell culture process in this study was conducted with reference to previous studies [2,3]. For the isolation and culture of primary hippocampal neurons, pregnant mice with embryos at 15.5 days gestation were euthanized. Following uterus extraction, the embryonic hippocampi were isolated, carefully de-membraned, and dissociated. The tissue was finely chopped, subjected to enzymatic treatment for 15 min, and centrifuged to obtain the supernatant. Prior to seeding, the MEA surfaces were functionalized with poly-D-lysine to promote cell attachment. Cells were then resuspended in

Neurobasal Plus medium and plated onto the MEA chip at a seeding density of 2,000 cells/mm^2, followed by incubation at 37°C in a CO_2 incubator. Medium was changed every 3 days.

Supplementary Text S3 Algorithm for neuronal firing synchrony coefficient.

To quantitatively assess the synchrony of neuronal action potential firing across different nodes, a sliding time window approach was employed to convert the spike event sequences recorded from each channel into binary vectors, where 0 represents the resting state and 1 indicates a firing event. The length of the time window was determined based on the average inter-spike interval of recording nodes, ensuring that the synchrony analysis was performed under a consistent and representative temporal scale. Subsequently, Pearson's correlation coefficient was used to evaluate the correlation between the binary vectors of different recording nodes, as defined in Equation 1:

$$c = \frac{\mathrm{cov}(X,Y)}{\sigma_X \sigma_Y} = \frac{\sum_{i=1}^{n}(X_i - \mu X)(Y_i - \mu Y)}{\sqrt{\sum_{i=1}^{n}(X_i - \mu X)^2}\sqrt{\sum_{i=1}^{n}(Y_i - \mu Y)^2}} \quad (1)$$

Here, X_i and Y_i denote the firing states (0 or 1) of different recording nodes within the ith time window, while μX and μY represent the mean firing probabilities of the corresponding recording nodes. A correlation coefficient closer to 1 indicates a higher degree of synchrony between the firing activities of the two recording nodes, whereas a value approaching 0 suggests weaker synchrony.

In this study, Pearson's correlation coefficients were systematically calculated among all node pairs using the above method to quantitatively characterize the firing synchrony between neurons. Finally, a synchrony heatmap was constructed to visualize the distribution of synchronization values, providing important insights into the functional connectivity patterns and dynamic properties of the neuronal network.

In addition to the second-level sliding windows determined by the mean inter-spike intervals, we also employed millisecond-level windows (e.g., 100 ms and 400 ms)—which are more conventional in network firing synchrony analysis—to re-evaluate the synchrony coefficients, functional connection counts, and network similarity metrics in Sections 3.3 and 3.5. The results across these varying temporal scales yielded consistent trends, further validating the robustness of our conclusions.

Supplementary Text S4 Network similarity analysis based on Levenshtein distance method

To quantitatively evaluate the functional connectivity similarity of networks across trials, we developed an analytical framework that integrates a sequence-encoding scheme with the Levenshtein distance method.

1. Sequence encoding based on the Louvain algorithm

The Louvain algorithm partitions active nodes into distinct functional subnetworks for each trial. Based on the community membership of these nodes, a representative connectivity sequence (V_{trial}) is developed to characterize the connectivity of the entire network:

$$V_{\text{trial}} = [\underbrace{n_{1,1}, n_{1,2}, \ldots,}_{subnetwork\_1} \underbrace{n_{2,1}, n_{2,2}, \ldots,}_{subnetwork\_2} \ldots, \underbrace{n_{k,1}, \ldots}_{subnetwork\_k}] \tag{2}$$

here, $n_{k,j}$ denotes the identifier of the i-th electrode belonging to the k-th subnetwork. This encoding format ensures that nodes with strong functional coupling are adjacently grouped, thereby enabling subsequent analyses to capture the dissolution, merging, or reconfiguration of these functional communities across different trials.

2. Quantification of network similarity

Subsequently, the Lenvenshtein ratio (R) between connectivity sequences of successive trials was calculated to quantify network similarity:

$$R = \frac{(|V_1| + |V_2|) - L}{(|V_1| + |V_2|)} \tag{3}$$

Here, $|V|$ represents the length of the sequence, L represents the Levenshtein edit distance between V_1 and V_2.

Given the stochastic nature of the community labels generated by the Louvain algorithm, we implemented a label alignment procedure by tracking persistent 'anchor nodes' to identify and match corresponding subnetworks across trials. This composition-based matching mechanism effectively eliminates the interference caused by arbitrary label switching, ensuring the consistency of network connectivity comparisons between different trials.

Supplementary Text S5 Algorithm for joint ISI distribution.

To quantify the influence of different stimulation patterns on the firing patterns of the in vitro biological neural network, this study performed a joint ISI distribution analysis across all effective electrode sites. The specific analysis details are as follows:

1. Matrix Initialization: Initialize a two-dimensional matrix, binCounts (X, Y), with all elements set to zero. The matrix dimensions are defined as X = Y = (MaxInterval - MinInterval) / Bin). This matrix is used to store the occurrence counts of different combinations of inter-spike time intervals.
2. This matrix will be used to store the counts of different time interval combinations.
3. For each spike sequence t[i], we calculate the current ISI (Interval_I = t[i] - t[i-1]) and the next consecutive ISI (Interval_I_Plus = t[i+1] - t[i]).
4. Subsequently, the index for the X-coordinate (binX) and Y-coordinate (binY) in binCounts are calculated for Interval_I and Interval_I_Plus, respectively, using the following formula: binX = (Interval_I - MinInterval) / Bin and binY = (Interval_I_Plus - MinInterval) / Bin.
5. Increment the value of the binCounts matrix at coordinates (binX, binY) by 1, i.e., binCounts[binX, binY] = binCounts[binX, binY] + 1.
6. The binCounts matrices from all individual electrode sites are then aggregated to generate the matrix, binCounts_trial.

7. Visualize the values in the binCounts_trial matrix on a graph using a color scale. Each matrix element's value determines the color displayed at the corresponding position.

By executing this algorithm, a statistical analysis of the time intervals between neural spike events can be performed, and the results are effectively visualized as a Joint ISI Distribution graph. This graph displays the frequency or count of different time interval combinations, thereby reflecting the burst tendency and firing patterns of network.

The parameters used in this study are as follows:

MaxInterval (sec) = 0.05

MinInterval (sec) = 0

Bin (sec) = 0.005

Bins per decade = 10

This configuration ensures the capture of representative inter-spike interval distributions within the population without compromising the global characteristics of network firing patterns. Furthermore, this parameterization provides an optimal balance between temporal resolution and statistical significance in multi-channel recordings.

Supplementary Text S6 Algorithm for network burst statistics.

In this study, a network-level burst detection approach was employed based on the MaxInterval method combined with a sliding time window, focusing on cross-nodes synchronous firing events. Specifically, a 100 ms sliding window was applied across all recording nodes, and a network burst was defined when at least three nodes exhibited spiking event within the same window. According to this criterion, the number of spikes within each burst was quantified, and for each recording node, the ratio of spikes occurring within bursts to the total number of spikes during the recording period was calculated.

This method provides a statistical framework for quantifying network-level burst activity and establishes a basis for further investigation of neuronal network synchrony and its dynamic properties.

References

1. Zhang, K.; Deng, Y.; Liu, Y.; Luo, J.; Glidle, A.; Cooper, J.M.; Xu, S.; Yang, Y.; Lv, S.; Xu, Z. Investigating Communication Dynamics in Neuronal Network Using 3D Gold Microelectrode Arrays. ACS nano 2024, 18, 17162–17174.
2. Ray, J.; Peterson, D.A.; Schinstine, M.; Gage, F.H. Proliferation, Differentiation, and Long-Term Culture of Primary Hippocampal Neurons. Proceedings of the National Academy of Sciences 1993, 90, 3602–3606.
3. Cano-Jaimez, M.; Tagliatti, E.; Mendonca, P.R.F.; Nicholson, E.; Vivekananda, U.; Kullmann, D.M.; Volynski, K.E. Preparation of Dissociated Mouse Primary Neuronal Cultures from Long-Term Cryopreserved Brain Tissue. Journal of Neuroscience Methods 2020, 330, 108452.
